# Sex/gender differences in autistic traits, intelligence and executive functions of school-aged autistic children without intellectual disability

**DOI:** 10.1007/s00737-025-01663-1

**Published:** 2026-01-07

**Authors:** Katarína Polónyiová, Peter Teličák, Klaudia Kyselicová, Dóra Dukonyová, Daniela Ostatníková

**Affiliations:** 1 Institute of Physiology, Academic Research Centre for Autism, Faculty of Medicine, Comenius University in Bratislava, Bratislava, Slovakia; 2 Department of Psychology, Faculty of Philosophy and Arts, University of Trnava, Trnava, Slovakia; 3 Incubator of Kinanthropology Research, Faculty of Sports Studies, Masaryk University, Brno, Czech Republic

**Keywords:** Autism spectrum disorder, Autistic traits, Intelligence, Executive functions

## Abstract

**Background:**

ASD has been more often diagnosed and researched in men than women, shaping diagnostic criteria which may not adequately capture the female presentation. Examining differences between girls and boys with ASD could enhance diagnostic accuracy and help reduce gender-related biases in research and clinical practice. The aim of this research was to analyze potential differences in autistic traits, intelligence, and executive functions of school-aged girls and boys diagnosed with ASD without intellectual disability.

**Methods:**

The research sample consisted of 79 children with ASD, 20 girls and 59 boys, aged between 6 and 12 years. Autistic traits were measured by Autism Diagnostic Observation Schedule – Second Version and Autism Diagnostic Interview-Revised, intelligence by the Woodcock-Johnson International Editions II, and executive functions by Wisconsin Card Sorting Test and Behavior Rating Inventory of Executive Function 2.

**Results:**

Girls scored lower in the amount of restricted, repetitive and stereotyped behaviors, but showed more severe deficits in Emotion Regulation, Cognitive Regulation and clinical scales Shift and Initiate, as measured by BRIEF-2.

**Conclusion:**

Our results indicate girls with ASD exhibit certain differences from boys with ASD, which may be diagnostically relevant and helpful for their early detection and access to necessary resources and support. Nevertheless, extensive further research on the sex/gender differences and female ASD presentation is still needed.

## Article highlights 3-5

ASD has been more often diagnosed and researched in men than women, potentially resulting in biased diagnostic criteria and female underdiagnosis.

There is still a considerable lack of research on the distinct traits and presentation of autism in females.

Girls in our research scored lower in the amount of restricted, repetitive and stereotyped behaviors, but showed more severe executive function deficits as measured by BRIEF-2.

Our results indicate that girls with ASD exhibit certain differences from boys with ASD, which may be diagnostically relevant and helpful for their early detection.

## Introduction

Autism spectrum disorder (ASD) is a pervasive neurodevelopmental disorder characterized by deficits in the ability to initiate and sustain reciprocal social interaction and communication and by a range of restricted, repetitive, and inflexible patterns of behavior, interests, or activities (World Health Organisation 2019). Epidemiological studies as well as clinical practice show that ASD is more often diagnosed in men than women, with current estimates between 3:1–4,3:1 across the spectrum (Brugha et al. 2016; Maenner et al. 2020), while true numbers might be closer to 2:1 or 3:1, given the assumed underdiagnosis of women (Loomes et al. 2017).

This ratio is significantly dependent on intelligence, 5.75:1 in individuals with IQ > 70, compared to the 1.9:1 in individuals with an intellectual disability (ID) (Kim et al. 2011), and is also varying in different age groups, as affected by significant delay in the mean age of referral and diagnosis for girls (Rutherford et al. 2016). While average age of concern occurs before 2 years of age for both girls and boys, average age of diagnosis in girls is 5 years or even later for those with higher verbal IQ (McDonnell et al. 2021). The estimated ratio may also be skewed by perception of ASD as a predominantly male disorder, held by many healthcare professionals and the general public, and by potential bias in standard diagnostic methods such as ADOS-2, which were developed based on the male autism phenotype (Adamou et al. 2018; Rosen et al. 2021).

Moreover, there are differences in frequent comorbidities which make ASD more apparent in boys, e.g. greater externalizing and behavioral problems such as aggressive behavior (Mandy et al. 2012), while girls often show less noticeable and internally experienced difficulties including anxiety and depression (De Giambattista et al. 2021). In the absence of additional intellectual or behavioral problems, girls are less likely to meet diagnostic criteria at equivalently high levels of autistic traits (Dworzynski et al. 2012). There is a large discussion on if the diagnostic criteria should be broadened to capture the presentations of women just below the threshold, especially not exceeding the cut-off in the restricted interests domain (Tsirgiotis et al. 2024).

Furthermore, girls without ID are often not even referred for diagnostic evaluation, due to a better ability of “masking” or “camouflaging” their autistic traits in various social situations with the intent to fit in, e.g. by thinking how to act next, preparing conversations in advance, making lists of prompts and prosocial responses or by talking with a less monotone voice (Day et al. 2024). Girls with ASD are typically better in imitation and social learning than boys (Corbett et al. 2021) and are more prone to realizing their differences and attempting to camouflage them in social situations as an adaptive mechanism (Tubío-Fungueiriño et al. 2021). However, camouflaging their autistic characteristics during clinical assessments, consciously or not, may lead to missed diagnosis and subsequent lack of appropriate support (Hull et al. 2020).

### Autistic traits

Despite growing interest in sex/gender differences in ASD, much remains uncertain about whether a distinct female profile of autistic traits exists and whether ASD is far more prevalent in males, or females express their autism in ways which do not always meet current diagnostic criteria. The existing literature on this topic is still insufficient to draw definitive conclusions and the findings are inconsistent and often influenced by biases in diagnosis and research sampling.

However, some sex/gender differences have been reported more consistently than others. Girls with ASD are typically better at reciprocal social interaction than boys (Backer Van Ommeren et al., 2017), have a wider range of facial expressions used to communicate and more imaginative play as measured by ADI-R (Beggiato et al. 2017). They also typically demonstrate less repetitive and stereotyped behaviors then boys (Natoli et al. 2023; Stephenson et al. 2023) even with male-equivalent levels of social and communication impairment (Mandy et al. 2012; Van Wijngaarden-Cremers et al. 2014), and have no significant differences in sensory interests and insistence on sameness and routine (Edwards et al. 2024).

Other studies found the differences to be more qualitative than quantitative, with the autistic girls’ interests being closer to those of neurotypical girls than to those of autistic boys, e.g. dolls, animals, horses, celebrities, fashion, make-up, arts, books, and manga (Bourson and Prevost 2024; Brown et al. 2024; Sutherland et al. 2017). In order to prevent the risk of misdiagnosis, diagnostic tools could benefit from the addition of more female-like examples.

### Intelligence

Sex/gender differences in ASD are also moderated by intelligence and while among individuals with ASD with ID, girls seem to be more severely affected than boys, in those without ID, girls present with fewer autistic traits (Saure et al. 2023), potentially contributing to the female underdiagnosis. Yet, the amount of research on sex/gender differences in broad cognitive profiles among children with ASD remains limited and yields inconsistent findings, highlighting the need for further investigation.

Previous research suggests that individuals with ASD typically show uneven cognitive profiles, in which the partial abilities comprising overall intellect can span over several standard deviations from each other (Billeiter and Froiland 2022), and form spiky performance profiles of strengths and weaknesses with weaker correlations between different cognitive domains (Goldstein et al. 2008). According to Wilson (2023), individuals with ASD score approximately one standard deviation lower in processing speed and also exhibit slightly reduced scores in working memory compared to the neurotypical group. Very little is known about whether there are some specific cognitive ability profiles typical for girls with ASD.

Limited amount of previous studies on this subject came to conflicting results. Girls with ASD often have lower IQ than boys, but similar adaptive functioning (Di Vara et al. 2024), which helps hide some of the deficits in their everyday lives. Other studies did not find any significant differences in overall IQ of girls and boys with ASD, with girls scoring lower in perceptual reasoning (Giofrè et al. 2024; Ji et al. 2023), but also noted that the observed sex/gender differences are comparable to the ones found in neurotypical populations.

### Executive functions

Executive functions (EFs) are a set of higher-order cognitive processes governing various cognitive, emotional and behavioral functions which are necessary for goal-directed behavior, (Gioia et al. 2002). Individuals with ASD regardless of their IQ often exhibit impairments in both verbal and visuospatial working memory (Seng et al. 2021; Wang et al. 2017), inhibition (Geurts et al. 2014), as well as difficulties with cognitive flexibility (Geurts et al. 2004). When analyzing more complex EFs, deficits in planning, verbal fluency (Czermainski et al. 2014), shifting/cognitive flexibility, inhibition, initiation and task-monitoring (Blijd-Hoogewys et al. 2014) are often present.

Furthermore, EFs are tightly interrelated with autistic traits (Burroughs et al. 2024; Ko et al. 2024), especially the ability to suppress interfering information (inhibition) and flexibly shift between patterns of responding (cognitive flexibility) seem to be protective against higher order restricted, repetitive behaviors, interests, and activities (Faja and Nelson Darling 2019; Iversen and Lewis 2021; Schmitt et al. 2018), particularly in boys (Bölte et al. 2011). EF deficits were also found to be related to greater reciprocal social interaction and communication deficits in girls with ASD (Torske et al. 2023).

Several studies found significant sex/gender differences in EFs of individuals with ASD, with girls with ASD demonstrating a significantly poorer inhibition (Lemon et al. 2011), more perseverative errors and fewer categories completed in WCST (Memari et al. 2013) and more difficulties with overall EFs as rated by their parents using the BRIEF-2 (White et al. 2017). However, some of these differences might not be specific to individuals with ASD, but rather attributed to sex/gender differences in general. Kiep and Spek (2017) found that adult women with ASD performed worse on the working memory and had more perseverations on the WCST than neurotypical women, but no specific impairments typical for men or women with ASD were found. Contrastingly, Demetriou et al. (2021) found that females with or without ASD had better processing speed, cognitive flexibility, verbal learning and semantic fluency, yet any sex/gender differences in individuals with ASD were similar to the ones in neurotypical group.

Although interest in sex/gender differences in autism has increased in recent years, there remains a significant lack of research examining these differences in key cognitive and behavioral domains, particularly in school-aged children without intellectual disability. Moreover, findings across studies remain inconsistent, and research examining multiple cognitive and behavioral domains within the same cohort remains limited.

The objective of this research was to investigate potential differences between school-aged girls and boys with ASD without intellectual disability, focusing on their autistic traits, intelligence, and executive functions.

## Methods

### Research sample

Our research sample consisted of 79 children with ASD, 20 girls and 59 boys, collected from September 2020 to September 2024. The female to male ratio was 3.95:1, which roughly reflects the prevalence of ASD across genders. The groups were of a balanced mean age and overall IQ. Their ages were between 6 and 12 years, with the mean age of girls 9.85 (SD = 1.57) and mean age of boys 9.12 (SD = 1.74). Their intelligence ranged from 72 to 132, with mean IQ of the total sample 99.41 (SD = 16.4). Girls had a mean IQ of 96.55 (SD = 13.00) and boys had a mean IQ of 100.37 (SD = 17.39).

All were diagnosed with ASD in our center and tested at the time of their first autism diagnostics. Participants were recruited through a university-affiliated autism research and diagnostic center operating under the Faculty of Medicine. The center provides comprehensive autism diagnostic services free of charge, as well as tailored professional guidance for families. In exchange, families consent to their child’s participation in research. Referrals for diagnostics were made by school psychologists, pediatricians, neurologists, psychiatrists, or directly by parents who suspect their child might have autism. All participants were diagnosed with ASD using ADOS-2 Module 3, as only children with fluent speech were able to understand the instructions of all selected tests. Children with syndromic autism or co-occurring conditions such as suspected development of psychotic disorder, selective mutism, epilepsy, or diagnosed ADHD were excluded from the sample. The study was approved by the Ethics Committee. Participation in the study was voluntary. The procedure was explained in detail to the parents and their children participated only after both parents signed the informed consent form.

### Measures

To diagnose the children with ASD and to measure their autistic traits, we used the standard combination of *Autism Diagnostic Observation Schedule – second version (ADOS-2)* (Lord et al. 2012) and *Autism Diagnostic Interview-Revised (ADI-R)*(Lord et al. 1994). Beyond the standardized ADOS-2 assessment, additional qualitative clinical observations were conducted by the first author to capture the specific content of children’s special interests, which were not systematically measured but noted during diagnostic sessions. *Woodcock-Johnson International Editions II (WJ IE)*(Ruef et al. 2003) was used to measure intelligence. It measures current overall intellectual performance and its three broad abilities: Verbal Ability, Thinking Ability, and Cognitive Efficiency and seven subtests Verbal Ability, Memory for Names, Spatial Relations, Sound Discrimination, Concept Formation, Visual Matching, Numbers Reversed, and Quantitative Concepts. Executive functions were measured by two methods: firstly a direct testing of children´s cognitive flexibility using *Wisconsin Card Sorting Test (WCST)*(Berg 1948) and secondly a parent-rated questionnaire *Behavior Rating Inventory of Executive Function*,* Second Edition (BRIEF-2)*(Gioia et al. 2015). The parent version of the BRIEF-2 consists of nine clinical scales (Inhibit, Self-Monitor, Shift, Emotional Control, Initiate, Working memory, Plan/Organize, Task-Monitor, Organization of Materials), from which three composite scores are calculated (Behavioral Regulation, Emotion regulation, Cognitive Regulation) a finally a Global Executive Composite representing the overall level of executive functioning. Higher scores indicate a greater degree of deficits in the respective areas. All instruments were chosen based on their established validity and age norms covering the 6–12-year range, ensuring that the measures remain developmentally appropriate and comparable across this span.

### Statistical analysis

Data were analyzed using SPSS and Jamovi software. Descriptive analysis was performed and skewness, kurtosis, (−2 - +2) (Byrne 2013; George and Mallery 2010; Tabachnick et al. 2013).

and Q-Q plots were used as reference points to assess the normality of the data distribution. A Student’s independent samples t-test, Welch’s t-test, or the Mann–Whitney U test was conducted, depending on whether the assumptions of normality and homogeneity of variances were met, to assess differences between boys and girls. Homogeneity of variances was tested using Levene’s test. Levene’s test. Effect sizes were calculated using Cohen’s d (for t-tests) and r (for the Mann–Whitney U test).

## Results

Descriptive statistics of all measured variables of autistic traits, intelligence and executive functions are presented in the Table 1.

**Table 1:** Descriptive statistics of the measured variables

	Mean		Median		Standard deviation		Skewness		Kurtosis	
Sex	b	g	b	g	b	g	b	g	b	g
Age	9.12	9.85	8.84	10.24	1.74	1.57	0.34	−0.53	−0.89	−0.89
**ADI-R**										
Qualitative Abnormalities in Reciprocal Social Interaction	14.3	15.15	14	14.50	4.74	5.71	0.62	0.22	−0.07	0.13
Qualitative Abnormalities in Communication	12.32	12.5	12	12.00	4.37	4.35	0.24	−0.07	−0.73	−1.14
Restricted Repetitive and Stereotyped Patterns of Behavior	5.90	5.10	6	5.50	2.4	1.83	0.38	0.07	−0.38	−1.48
Abnormality of Development Evident at or Before 36 Months	2.66	3.25	3	3.00	1.70	1.41	−0.06	−0.49	−1.14	−0.02
**ADOS-2**										
Language and Communication	6.64	6.20	7	6.50	3.26	2.31	−0.02	−0.07	−0.42	−0.77
Reciprocal Social Interaction	11.49	11.75	11	11.50	4.22	3.58	0.31	−0.32	−0.55	−0.69
Imagination/Creativity	1.22	1.30	1	1.00	0.65	0.66	−0.24	−0.40	−0.62	−0.55
Stereotyped Behaviors and Restricted Interests	3.71	2.60	3	2.00	1.52	1.39	0.48	0.68	−0.76	0.05
Other Abnormal Behaviors	1.32	0.90	1	1.00	1.17	0.79	0.62	0.90	−0.18	1.39
Social Affect	10.86	10.75	10	11.00	4.30	3.40	0.37	−0.26	−0.79	−0.66
Restricted, Repetitive and Stereotyped Patterns of Behavior	4.00	2.80	4	2.50	1.63	1.47	0.25	0.60	−0.86	−0.43
General severity score	14.86	13.60	15	14.00	4.53	4.2	0.46	−0.45	−0.21	−0.15
**WJ-IE**										
General intellectual ability	100.37	96.55	100.00	95.00	17.39	13.00	−0.33	0.16	−0.19	−0.55
Verbal Ability	100.39	93.25	105.00	92.50	18.66	11.64	−0.73	0.18	−0.34	0.59
Thinking Ability	102.88	100.35	107.00	95.50	19.52	16.80	−1.85	0.45	2.16	−1.04
Cognitive Efficiency	93.63	93.85	96.00	96.00	15.91	10.36	−0.27	−1.16	0.27	2.9
Memory for Names	103.17	102.05	103.00	98.00	13.95	16.61	0.10	−0.08	−0.62	−1.06
Spatial Relations	102.86	97.40	104.00	95.00	14.17	11.46	−0.53	−0.39	1.40	−0.89
Sound Patterns	103.44	102.00	104.00	106.50	18.18	20.5	−0.19	−0.39	−0.86	−0.28
Concept Formation	104.86	99.35	107.00	95.00	15.71	18.41	−0.38	0.35	−0.06	−0.56
Visual Matching	89.12	89.35	91.00	92.50	16.9	19.38	−0.17	−0.19	−0.61	1.4
Numbers Reversed	96.32	94.30	98.00	96.00	16.33	17.14	−0.42	−1.63	0.74	3.81
Quantitative Concepts	103.42	94.15	105.00	93.00	20.48	10.17	−0.03	0.64	−0.43	−0.24
**BRIEF-2**										
Inhibit	64.47	69.00	64.00	72.00	11.29	11.95	−0.14	−0.70	−1.00	−0.15
Self-Monitor	65.41	68.00	63.00	69.00	8.76	9.23	−0.30	−0.20	−0.62	−1.36
Shift	69.76	75.90	69.00	75.50	11.35	10.39	−0.04	−0.19	−0.49	−1.37
Emotional Control	68.02	72.55	66.00	75.00	9.32	8.20	−0.01	−0.91	−0.40	0.48
Initiate	61.36	67.10	63.00	66.00	9.50	11.16	−0.22	−0.23	−0.02	−0.49
Working memory	63.03	65.85	64.00	66.00	11.69	10.72	−0.19	−0.36	−1.11	−0.31
Plan/Organize	60.15	63.75	60.00	69.00	9.70	11.14	−0.24	−0.80	−0.75	−0.65
Task-Monitor	60.02	64.50	62.00	63.00	9.99	8.61	−0.35	−0.12	−1.21	−0.61
Organization of Materials	60.51	60.25	63.00	61.00	10.35	9.77	−0.27	−0.03	−0.93	−0.44
Behavioral Regulation	65.86	70.70	64.00	73.00	10.32	9.69	−0.12	−0.97	−0.98	1.18
Emotion Regulation	70.14	76.50	71.00	76.00	9.95	9.34	−0.01	−0.20	−0.28	−0.66
Cognitive Regulation	61.86	67.75	62.00	66.50	9.36	10.81	−0.17	−0.29	−0.83	−0.22
Global Executive Composite	68.90	71.75	69.00	71.50	10.49	9.29	−0.16	−0.81	−0.88	0.61
**WCST**										
Errors	26.63	23.60	28.00	23.00	10.66	11.2	−0.08	0.48	−0.66	0.24
Perseverative errors	11.75	8.45	11.00	7.50	7.91	6.33	0.85	0.46	1.19	−0.31
Mean reaction time	3735.65	3303.13	3395.18	2725.19	1658.38	1891.36	1.50	3.08	1.38	1.12

g = girls, b = boys, Other Abnormal Behaviors = hypo/hyperactivity, disruptive behaviors, anxiety

Table 2 presents the tested parameters from Levene’s test for each variable. The results of the homogeneity of variances test indicate acceptable variance homogeneity for all variables except for Other Abnormal Behaviors (ADOS-2) and Verbal Ability (WJ). For these variables, we applied Welch’s t-test to account for unequal variances.

**Table 2:** The results of levene’s test of the measured variables

	F	df	df2	*p*
Age	0.47	1	77	0.493
**ADI-R**				
Qualitative Abnormalities in Reciprocal Social Interaction	0.72	1	77	0.398
Qualitative Abnormalities in Communication	0.01	1	77	0.933
Restricted Repetitive and Stereotyped Patterns of Behavior	0–00	1	77	0.996
Abnormality of Development Evident at or Before 36 Months	2.08	1	77	0.153
**ADOS 2**				
Language and Communication	3.45	1	77	0.067
Reciprocal Social Interaction	0.92	1	77	0.339
Imagination/Creativity	0.12	1	77	0.732
Stereotyped Behaviors and Restricted Interests	0.26	1	77	0.609
Other Abnormal Behaviors	7.27	1	77	0.009
Social Affect	1.76	1	77	0.188
Restricted Repetitive and Stereotyped Patterns of Behavior	0.48	1	77	0.489
General severity score	0.57	1	77	0.451
**WJ**				
General intellectual ability	2.67	1	77	0.107
Verbal Ability	7.62	1	77	0.007
Thinking Ability	0.06	1	77	0.811
Cognitive Efficiency	5.02	1	77	0.058
Memory for Names	1.86	1	77	0.176
Spatial Relations	0.27	1	77	0.604
Sound Patterns	0.06	1	77	0.811
Concept Formation	1.22	1	77	0.271
Visual Matching	0.23	1	77	0.636
Numbers Reversed	0.05	1	77	0.826
Quantitative Concepts	10.40	1	77	0.052
**BRIEF-2**				
Inhibit	0.07	1	77	0.934
Self-Monitor	0.16	1	77	0.688
Shift	0.02	1	77	0.903
Emotional Control	1.31	1	77	0.257
Initiate	1.22	1	77	0.273
Working memory	1.34	1	77	0.250
Plan/Organize	1.39	1	77	0.242
Task-Monitor	2.07	1	77	0.154
Organization of Materials	0.81	1	77	0.370
Behavioral Regulation	1.15	1	77	0.287
Emotion Regulation	0.05	1	77	0.830
Cognitive Regulation	0.03	1	77	0.868
Global Executive Composite	1.56	1	77	0.215
**WCST**				
Errors	0.03	1	77	0.855
Perseverative Errors	0.45	1	77	0.505
Mean reaction time	0.32	1	77	0.572

Next, we tested for potential differences between boys and girls, as can be seen in Table 3. The results of the independent samples t-test indicated a significant difference in autistic traits, specifically in Restricted, Repetitive, and Stereotyped Patterns of Behavior domain, between boys (M = 4.00) and girls (M = 2.80), t (77) = 2.28, *p* = 0.005, d = 0.75, with girls scoring significantly lower. Similarly, a significant difference was found in Stereotyped Behaviors and Restricted Interests belonging under this domain, with girls (M = 2.60), t(77) = 2.44, *p* = 0.005, d = 0.75 scoring lower than boys (M = 3.71).

**Table 3:** The results of independent samples t-test for each individual variable

						95% CI		
	Statistic	df	*p*		Mean difference	Lower	Upper	Cohen´s d
Age	−1.67	77	0.099		−0.73	−1.61	0.14	−0.43
**ADI-R**								
Qualitative Abnormalities in Reciprocal Social Interaction	−0.86	77	0.391		−1.12	−3.69	1.46	−0.22
Qualitative Abnormalities in Communication	0.24	77	0.810		0.27	−1.98	2.52	0.06
Restricted Repetitive and Stereotyped Patterns of Behavior	1.97	77	0.125		0.80	−0.23	1.82	0.40
Abnormality of Development Evident at or Before 36 Months	−1.40	77	0.167		−0.59	−1.43	0.25	−0.36
**ADOS-2**								
Language and Communication	0.56	77	0.575		0.44	−1.13	2.02	0.16
Reciprocal Social Interaction	−0.25	77	0.807		−0.26	−2.36	1.84	−0.06
Imagination/Creativity	−0.48	77	0.636		−0.08	−0.41	0.25	−0.12
**Stereotyped Behaviors Restricted Interests**	**2.44**	**77**	**0.005**		**1.11**	**0.34**	**1.88**	**0.75**
Other Abnormal Behaviors*	1.81	48.86	0.079		0.42	−0.14	0.98	0.39
Social Affect	0.11	77	0.910		0.11	−1.89	2.12	0.02
**Restricted**,** Repetitive and Stereotyped Patterns of Behavior**	**2.28**	**77**	**0.005**		**1.20**	**0.38**	**2.02**	**0.75**
General severity score	1.11	77	0.271		1.44	−1.00	3.54	0.29
**WJ**								
General intellectual ability	0.90	77	0.371		3.29	−4.64	12.28	0.23
Verbal Ability*	2.21	53.27	0.059		7.14	−1.72	15.99	0.42
Thinking Ability	0.52	77	0.603		2.14	−7.12	12.18	0.14
Cognitive Efficiency	−0.06	77	0.954		−0.22	−7.81	7.37	−0.02
Memory for Names	0.23	77	0.769		1.12	−6.41	8.67	0.08
Spatial Relations	1.79	77	0.123		5.44	−1.52	12.45	0.40
Sound Patterns	0.30	77	0.766		1.07	−8.17	11.05	0.08
Concept Formation	1.82	77	0.198		5.44	−2.94	13.97	0.34
Visual Matching	−0.05	77	0.958		−0.23	−8.97	8.51	−0.01
Numbers Reversed	0.47	77	0.638		2.02	−6.50	10.54	0.12
Quantitative Concepts	1.99	77	0.056		9.37	−0.25	18.79	0.50
**BRIEF-2**								
Inhibit	−1.53	77	0.131		−4.53	−10.43	1.38	−0.40
Self-monitor	−1.13	77	0.262		−2.59	−7.17	1.98	−0.29
**Shift**	**−2.13**	**77**	**0.036**		**−6.14**	**−11.87**	**−0.41**	**−0.55**
Emotional Control	−1.94	77	0.057		−4.53	−9.20	0.13	−0.50
**Initiate**	**−2.23**	**77**	**0.028**		**−5.74**	**−10.86**	**−0.62**	**−0.58**
Working memory	−0.95	77	0.345		−2.82	−8.72	3.09	−0.25
Plan/Organize	−1.38	77	0.172		−3.60	−8.79	1.60	−0.36
Task-Monitor	−1.79	77	0.077		−4.48	−9.47	0.50	−0.46
Organization of Materials	0.10	77	0.922		0.26	−5.00	5.52	0.03
Behavioral Regulation	−1.84	77	0.070		−4.84	−10.07	0.40	−0.48
**Emotion Regulation**	**−2.51**	**77**	**0.014**		**−6.36**	**−11.42**	**−1.31**	**−0.65**
**Cognitive Regulation**	**−2.34**	**77**	**0.022**		**−5.89**	**−10.90**	**−0.87**	**−0.60**
Global Executive Composite	−1.08	77	0.284		−2.85	−8.11	2.41	−0.28
**WCST**								
Errors	1.09	77	0.280		3.03	−2.51	8.57	0.28
Perseverative errors	1.69	77	0.096		3.58	−0.60	7.19	0.44
Mean reaction time**	**Group** boys girls	**MR** 42.46 32.75			**U** 445.00	**p** 0.102	**r** 0.24	

^* =^ Welch’s t-test; ^* *=^ Mann Whitney U test

We found no differences in broad intellectual abilities or any of the subtests. When comparing the executive functions, we found no significant differences in any of the parameters of a direct measurement using WCST. The results further indicated the presence of significant differences between boys and girls in executive functions measured by BRIEF-2, specifically in clinical scales Shift, boys (M = 66.76) and girls (M = 75.90), t(77) = −2.13, *p* = 0.036, d = −0.55; and Initiate, boys (M = 61.36) and girls (M = 67.10), t(77) = −2.23, *p* = 0.028, d = −0.58; as well as the composite scores Emotion Regulation, boys (M = 70.14) and girls (M = 76.50), t(77) = −2.51, *p* = 0.014, d = −0.65, and Cognitive Regulation, boys (M = 61.86) and girls (M = 67.75), t(77) = −2.34, *p* = 0.028, d = −0.60. Statistically significant results are highlighted in bold in Table 3 below.

## Discussion

Historically, ASD has been diagnosed and studied predominantly in male populations, leading to the diagnostic criteria and tools which may not fully account for female presentation, resulting in female underdiagnosis. Investigating potential differences in autistic traits, intelligence, and EFs among girls and boys with ASD could be beneficial for reducing the related bias in ASD research, diagnostics and clinical practice.

### Autistic traits

Although research in this area remains scarce, current findings suggest the existence of a female-typical autistic traits presentation (Hull et al. 2020). We found girls to score significantly lower in the amount of restricted, repetitive and stereotyped patterns of behavior, in accordance with the majority of previous research (Beggiato et al. 2017; Mandy et al. 2012; Natoli et al. 2023; Stephenson et al. 2023; Van Wijngaarden-Cremers et al. 2014).

Qualitative clinical observations complemented the ADOS-2 assessment and highlighted differences in the content of special interests between girls and boys. Although the ADOS-2 assesses the intensity and potential impact of special interests rather than their qualitative nature, several girls in our sample exhibited special interests that are more traditionally girl-like, e.g. arts and crafts, hairstyles and fashion, or specific animals like foxes, consistent with findings from previous studies (Bourson and Prevost 2024; Brown et al. 2024; Sutherland et al. 2017). Such special interests could have been missed by a less skilled diagnostician and were often previously seen as normal by the parents, despite their pervasiveness.

Conversely, unlike many previous researchers (Backer Van Ommeren et al., 2017; Beggiato et al. 2017), we did not find any sex/gender differences in reciprocal social communication and interaction as measured by ADOS-2 or ADI-R. However, these results must be interpreted with careful consideration of the fact that due to male ASD bias or better camouflaging, many girls with less visible deficits in reciprocal social communication and interaction are never referred for diagnostic evaluation, and might be missing in our research sample, too.

### Intelligence

Due to a limited number of studies on this topic, sex/gender differences in the broad cognitive profiles of children with ASD remain an important area for future research. In our study, we found no differences in broad cognitive functions of girls and boys with ASD, which could be interpreted in multiple ways. Firstly, both groups had a comparable overall intelligence, in the IQ range between 70 and 130, and in such group intellectual differences might not be as distinctive.

Secondly, the fact that we found no differences could indicate that girls and boys with ASD have very similar cognitive profiles. There is a strong scientific consensus that compared to neurotypical children, children with ASD tend to have more uneven cognitive profiles with greater disparities between subtest scores (Billeiter and Froiland 2022), weaker correlations between different cognitive domains creating a more fragmented intellectual profile (Goldstein et al. 2008) and specific patterns of typical strengths and weaknesses (Wilson 2023).

However, since we only compared the girls and boys with ASD in this study, we can conclude that the cognitive profiles of both groups were similar, but further studies are needed to compare them to neurotypical girls and boys.

### Executive functions

Previous studies are inconsistent in their findings about possible sex/gender differences in EFs of individuals with ASD. Several studies found girls with ASD to have a significantly poorer inhibition (Lemon et al. 2011), cognitive flexibility (Memari et al. 2013), and overall EF deficits (White et al. 2017), but others propose there are no specific strengths or deficits typical for girl or boys with ASD (Demetriou et al. 2021; Kiep and Spek 2017).

Nonetheless, identifying the autistic female-typical profile of EFs, should it exist, could enhance the detection of girls with suspected ASD. ASD is particularly challenging to diagnose in females, due to camouflaging or possible differences in autistic traits presentation and a lack of awareness or experience with these specifics in some healthcare professionals. Contrastingly, EF deficits are more readily observed and evaluated by both parents and teachers, so finding these potential female-typical differences could lead to earlier identification and appropriate referral for differential diagnostics.

In our research, we found no sex/gender differences in EFs as measured directly by WCST. The test is known for being very useful for distinguishing children with or without ASD (Eigsti 2011); Memari et al. (2013) also found girls to have more perseverative errors and fewer categories completed. In our research sample, both girls and boys scored high in the amount of errors and perseverative errors, showing comparable degree of deficits.

Conversely, according to the parent-rated questionnaire BRIEF-2, girls in our research sample showed more severe deficits in Emotion Regulation composite score a clinical scale Shift belonging under it, as well as in Cognitive Regulation Composite score and a clinical scale Initiate. As these measure EFs which are among the most typically deficient in children with ASD (Blijd-Hoogewys et al. 2014; Gioia et al. 2015), our results suggest the girls with ASD are even more severely affected.

It is, however, noteworthy that while direct measures of executive functions did not reveal sex/gender differences, parental questionnaires did. This divergence may reflect a bias in parental reporting, where parents expect greater cognitive and emotional regulation from daughters than sons, leading to more critical assessments of girls.

###  Limitations and future research directions

The primary limitation of this study is its small research sample size. However, this sample includes all girls without intellectual or speech impairments who were diagnosed with ASD in our center over a four-year period, which further highlights the persistent lack of awareness about ASD in females and their general underdiagnosis in our country.

The absence of a neurotypical comparison group represents another limitation of our study. Future research should aim to include larger and more diverse samples, including neurotypical children, but also individuals who are near-diagnostic thresholds for ASD, particularly girls. Recruiting such cohorts is particularly challenging, but could provide necessary data about possible female-typical autistic traits representation, which does not meet current diagnostic criteria. A lack of research data on the female ASD phenotype is a major research obstacle, and subsequently contributes to biases in assessment tools and diagnostic practices (Kreiser and White 2014) and assumed female underdiagnosis.

Despite these constraints and given a lack of research on this topic, our findings aim to provide valuable insights for future research as well as for professionals working with autistic youth, individuals with ASD and their families. Our results suggest that girls with ASD exhibit certain differences from boys with ASD, which may be diagnostically relevant and could contribute to improved early detection strategies. Girls in our study demonstrated fewer repetitive and stereotyped behaviors, but experienced greater executive function impairments, especially as rated by their parents.

Nonetheless, extensive further research on the sex/gender differences and female ASD representation is still needed and essential for improving the early identification, accurate diagnosis, and increased availability of appropriate resources and therapeutic services for all girls and women with ASD.

## Data Availability

The data collected in this study will be obtainable from the corresponding author upon reasonable request.
